# Global vs local modularity for network community detection

**DOI:** 10.1371/journal.pone.0205284

**Published:** 2018-10-29

**Authors:** Shi Chen, Zhi-Zhong Wang, Liang Tang, Yan-Ni Tang, Yuan-Yuan Gao, Hui-Jia Li, Ju Xiang, Yan Zhang

**Affiliations:** 1 Neuroscience Research Center & Department of Basic Medical Sciences, Changsha Medical University, Changsha, Hunan, China; 2 South City College, Hunan First Normal University, Changsha, Hunan, China; 3 Department of Information Science and Engineering, Changsha Medical University, Changsha, Hunan, China; 4 School of Management Science and Engineering, Central University of Finance and Economics, Beijing, China; 5 School of Information Science and Engineering, Central South University, Changsha, China; Universitat Rovira i Virgili, SPAIN

## Abstract

Community structures are ubiquitous in various complex networks, implying that the networks commonly be composed of groups of nodes with more internal links and less external links. As an important topic in network theory, community detection is of importance for understanding the structure and function of the networks. Optimizing statistical measures for community structures is one of most popular strategies for community detection in complex networks. In the paper, by using a type of *self-loop* rescaling strategy, we introduced a set of global modularity functions and a set of local modularity functions for community detection in networks, which are optimized by a kind of the self-consistent method. We carefully compared and analyzed the behaviors of the modularity-based methods in community detection, and confirmed the superiority of the local modularity for detecting community structures on large-size and heterogeneous networks. The local modularity can more quickly eliminate the *first-type* limit of modularity, and can eliminate or alleviate the *second-type* limit of modularity in networks, because of the use of the *local* information in networks. Moreover, we tested the methods in real networks. Finally, we expect the research can provide useful insight into the problem of community detection in complex networks.

## Introduction

Community structures are ubiquitous in various complex networks, examples including the biological networks, social networks and technological networks [1]. This means that the networks generally consist of communities (or modules) with dense internal connections and sparse external connections. Generally, the communities (or modules) in networks are closely related to functional units in real-world networks, such as cycles and pathways in metabolic networks and protein complexes in the protein-protein interaction networks [1, 2], and they may have quite different topological properties from those at the level of the entire networks [2–5] and affect the dynamics in the networks[6]. Therefore, identifying the communities is of importance for understanding the structures and functions of the networks.

As an important topic in network theory, many methods have been proposed for detecting community structures in the networks based on various approaches. For example, some methods are based on similarity measures [7], some methods make use of dynamics on networks such as random walk dynamics [8, 9] and label propagation [10–12], while some methods are based on statistical models [13, 14] (see refs [1, 15, 16] for reviews). Especially, many of popular community-detection methods generally consist of the optimization of quality functions [1, 17–19]. For example, the famous Newman-Girvan modularity (Mod) [20] can be used as an objective way to estimate the quality of community partitions, and thus it also implies a type of community-detection strategy, i.e., modularity optimization. Indeed, community detection can be regarded to be one kind of optimization problem, given the quality functions for evaluating community structures. Therefore, optimizing the quality functions has been one of the most popular strategies for community detection in complex networks[13, 20–23].

Modularity optimization has become a kind of popular way to discover communities in complex networks, while the original modularity has the resolution limit--some (small-size) communities may not be detected in large-size networks, even if communities are very obvious [24–26]. Specifically, communities will be merged if the inequality of resolution *k*_*s*_*k*_*t*_<2*M*⋅*e*_*st*_ is satisfied, where *k*_*s*_ and *k*_*t*_ are the total degrees of communities, *e*_*st*_ is the number of links between the communities, and *M* is the total number of links in the network. To avoid confusion with the latter, we called it as the first-type limit of resolution. Many improvement strategies as well as its variants have been proposed to deal with the resolution limit. For example, the edge re-weighting is an interesting strategy for enhancing community-detection methods [27–30]. In general, by assigning different weight to intra- and inter-community edges, community structure becomes more obvious, especially this will lead the (relative) decrease of the number (*e*_*st*_) of links between communities, and thus the resolution limit can be eliminated or alleviated. Recently, by focusing on the related shortcomings of modularity, a variant of modularity called modularity density was proposed, by adding two components (split penalty and community density) into original modularity [31–33]. The introduction of community density is helpful for eliminating the above resolution limit, while the split penalty can prevent excessive splitting of communities. The above approaches can improve the resolution of modularity, but it is not easy to adjust the resolution of modularity. Another kind of more simple and effective approach to this resolution limit is to add a resolution parameter into the definitions of the original modularity directly or indirectly, leading to the multi-resolution modularity [13, 34–37]. By adjusting the resolution parameter, communities of different sizes can be identified, and thus the resolution limit is naturally resolved. Different from the former approaches, adding the resolution parameter is equivalently to vary the background of communities to change the resolution of modularity. However, the multi-resolution modularity may encounter another problem--with the increase of resolution parameter, (large-size) communities may split into small parts before all (small-size) communities are revealed [37, 38]. We called this phenomenon as the second-type limit of resolution. Moreover, according to different concerns, there are many other extended definitions of modularity. For example, an alternative way of defining the resolution parameter in multilayer modularity was introduced in [39], while other extensions of modularity were proposed to deal with directed networks [40], weighted networks [41], signed networks [42], and overlapping communities [43].

As we know, modularity is defined generally by evaluating the fraction of links within communities minus the expected values in the null model [13, 20, 21]. The null model is crucial, which affects the definitions of modularity and the results in community detection. There existed several classical choices of the null models, such as the configuration model as well as the Erdös-Rényi model [13, 44–46]. Traditional modularity functions using these null models are generally called global modularity, because the null models are based on the assumption of the *global connectivity* of communities in the networks, that is, the connections between all pairs of nodes are possible. Previous studies have shown that the global modularity is easily to encounter the first-type resolution limit, and even by using its multi-resolution version, it may still encounter the second-type limit [37, 44]. Interestingly, many networks have communities (or modules) that are linked only with a small number of communities. This phenomenon can be called the *local connectivity* of communities in the networks. Modularity functions that take into account this information may provide a view of different depth into the community structures in the networks. Generally, this type of modularity functions can therefore be called as local modularity [47, 48]. Differently from the global modularity, different communities are generally assigned different backgrounds in local modularity. It has been shown that this enables local modularity to tolerate the above resolution limits better than global modularity [47].

Recently, based on the general self-loop rescaling strategy, we developed one uniform framework for the multi-resolution modularity [46]. The self-loop rescaling strategy has several advantages. (a) By assigning a self-loop (self-link) to each vertex, the resolution of modularity can be adjusted easily to identify communities at different levels. Because, for example, positive self-loop can increase (inner) degrees of communities (or say link density within communities), but does not change the link density between communities. This will increase the difference between the intra- and inter-community link-densities, leading that the communities can be disconnected more easily. From another viewpoint, this will increase the relative sizes of communities, leading that modularity can escape from the resolution limit (see example for analysis in Appendix). (b) The self-loop rescaling strategy can control the formation of the null model easily, and thus various (multi-resolution) modularity, including local modularity, can be derived based on the original modularity [34, 44, 46]. (c) The derived modularity by the self-loop rescaling can be maximized by existing modularity optimization algorithms [17, 49, 50], which can extend the application of the existing algorithms.

In this paper, as an extension of our previous works, we firstly introduce two sets of modularity functions for community detection in complex networks, including two global modularity functions and four local modularity functions respectively, by the self-loop rescaling strategy. By a kind of the self-consistent method for optimizing modularity, the modularity functions are applied to community detection. We evaluate the performance of the modularity and carefully compare their behaviors in community detection. The results confirm the superiority of the local modularity in detecting community structures on large-size and heterogeneous networks.

## Methods

### Global and local modularity for community detection

For a given community division in a network, the mathematical form of generalized (multi-resolution) modularity is denoted by

$$\begin{array}{l} Q=\frac{1}{2M}\sum_{i,j}\left(A_{ij}-\gamma \frac{k_{i}^{eff}k_{j}^{eff}}{2M_{C_{i}}^{eff}}\right)\delta (C_{i},C_{j})\\ \;=\frac{1}{2M}\sum_{s}\left(k_{s}^{in}-\gamma \frac{{\left(k_{s}^{eff}\right)}^{2}}{2M_{s}^{eff}}\right) \end{array},$$ (1)

where *γ* is a tunable resolution parameter; *A*_*ij*_ is the adjacent matrix of the network (*A*_*ij*_ =1 if there exists a link between nodes *i* and *j*, and zero otherwise); *C*_*i*_ is the community to which node *i* belongs; the Kronecker delta function *δ*(*C*_*i*_,*C*_*j*_) = 1 if nodes *i* and *j* belong to the same community, and zero otherwise; *M* = ∑_*ij*_*A*_*ij*_/2 is the number of links in network; $k_{s}^{in}$ is the inner degree of community *s*; $k_{i}^{eff}$ is the *effective* degree of node *i* in the null model (e.g., it is the degree of node in the CM-based model), while $k_{s}^{eff}=\sum_{}k_{i}^{eff}\cdot \delta (C_{i},s)$, which is the sum of the *effective* degree of nodes in community *s*, denotes the *effective* total degree of community *s*, and $2M=\sum k_{s}^{eff}$; $M_{s}^{eff}$ denotes the *effective* number of links that is related to community *s* in the null model. Please see Table 1 for different formations of $k_{i}^{eff}$ and $M_{s}^{eff}$, by which the definitions of modularity are determined.

**Table 1:** Various definitions of global and local modularity. *M*_Ω(*s*)_ and $\bar{M_{\Omega (s)}}=\bar{k}\cdot N_{\Omega (s)}/2$ denote the number of links and the mean number of links in community *s* and the neighborhood of it, where $\bar{k}$ denotes the mean degree of network and *N*_Ω(*s*)_ denotes the number of nodes in community *s* and the neighborhood of it. Please refer to Methods section for *γ*_*s*_.

*Modularity*		$M_{s}^{eff}$	$k_{i}^{eff}$	$k_{s}^{eff}$	*Self-loop rescaling*
*Global*	$Q_{CM}^{\left(g\right)}$	*M*	*k*_*i*_	*k*_*s*_	*γ*_*s*_⋅*k*_*i*_−*k*_*i*_
	$Q_{ER}^{\left(g\right)}$	*M*	$\bar{k}$	$\bar{k_{s}}$	$\gamma_{s}\cdot \bar{k}-k_{i}$
*Local*	$Q_{CM}^{\left(l\right)}$	*M*_Ω(*s*)_.	*k*_*i*_	*k*_*s*_	*γ*_*s*_⋅*k*_*i*_−*k*_*i*_
	$Q_{ER}^{\left(l\right)}$	*M*_Ω(*s*)_	$\bar{k}$	$\bar{k_{s}}$	$\gamma_{s}\cdot \bar{k}-k_{i}$
	$Q_{CM}^{\left(<l>\right)}$	$\bar{M_{\Omega (s)}}$	*k*_*i*_	*k*_*s*_	*γ*_*s*_⋅*k*_*i*_−*k*_*i*_
	$Q_{ER}^{\left(<l>\right)}$	$\bar{M_{\Omega (s)}}$	$\bar{k}$	$\bar{k_{s}}$	$\gamma_{s}\cdot \bar{k}-k_{i}$

Here, we introduce two sets of modularity functions, which include two *global* modularity functions and four *local* modularity functions respectively (see Eq (1) and Table 1 for definitions). The null model of modularity is critical to the definition of modularity, where the form of $M_{s}^{eff}$ is the most important factor of determining the difference between local and global modularity. For *global modularity*, $M_{s}^{eff}=M$, i.e., the links in whole network are considered, and for *local modularity*, $M_{s}^{eff}$ is determined by the neighborhood of community *s*, when estimating the probability of edge between vertices in random graphs under *certain constraints*, i.e. in null model. The more locally the communities of a network are connected with the rest of the network (that is, the *local connectivity* of communities is more apparent), the more obvious the difference between local and global modularity is. Conversely, $M_{s}^{eff}$ will be equal to *M*, and *γ*_*s*_ will be equal to *γ* when all communities in a network have *global community connectivity*, that is, all communities directly connect each other. In this case, the local modularity degenerates into the global modularity. The basic null models have two choices: the configuration null model (CM) and the Erdös-Rényi null model (ER). Because CM considers the heterogeneity of degree, while ER only uses the mean degree of node, we use CM and ER to denote the modularity with $k_{i}^{eff}=k_{i}^{}$ and $k_{i}^{eff}=\bar{k}$.

The equivalent (multi-resolution) *modularity* can also be constructed by the self-loop rescaling strategy (see Appendix and Table 1), because the modularity is affected by the network structure, the community division and the null model. The self-loop rescaling strategy can indirectly affect the null model and its weight in the modularity. The derived multi-resolution modularity can be maximized by the existing modularity optimization algorithms, which clearly extend the application of the algorithms.

Modularity optimization is a popular method for discovering communities in networks. However, according to previous studies, modularity (with fixed resolution parameter) cannot disconnect some (small-size) communities when the size of a network is very large, even if they are cliques [26]. Take the global CM-based modularity (note that it is equal to the Newman-Girvan modularity if *γ* = 1) as example, communities will be merged when *k*_*s*_*k*_*t*_<2*M*⋅*e*_*st*_/*γ*, where *k*_*s*_ and *k*_*t*_ are the total degrees of communities, *e*_*st*_ is the number of links between the communities, and *M* is the total number of links in the network [46]. The phenomenon is also called as the first-type limit of resolution. The problem above can be resolved by adjusting the resolution parameter, because with the increase of *γ*, the critical degrees of communities in the above inequality decrease gradually, and thus more (small-size) communities can be discovered. But, the modularity may still encounter another problem----with the increase of *γ*, some (large-size) communities may begin to split into some small parts before small-size communities are revealed completely [37, 38]. This is what we call the second-type limit of resolution. Compared to global modularity, the use of local information in local modularity may be able to improve the second-type limit of resolution.

### Network data

To compare the behaviors of the modularity optimization in detecting communities in the networks, we will apply the *global* and *local* modularity to a set of classical artificial networks with community structures (Lancichinetti-Fortunato-Rachicchi (LFR) networks) and a set of real-world networks. The LFR model has tunable sizes of networks and considers the heterogeneity in realistic networks [51]. In the networks, there are several parameters.

1. *N* denotes the number of nodes in the networks.
2. *k*_m_ and *k*_max_ denote the mean degree and maximum degree respectively.
3. *c*_min_ and *c*_max_ denote the minimum and maximum community sizes respectively.
4. *t*_1_ and *t*_2_ are respectively the power-law exponents of the distribution of degrees and community sizes.
5. *μ* denotes the mixing parameter which determines the ratio of the external degree of each node to the total degree of the node with respect to its community.

For real data, the real-world networks used in the study include the karate club network [52], polbooks network(http://www-personal.umich.edu/~mejn/netdata/), Football[53], the dolphin network[54], and Yeast [55].

## Results

The difference between the local and global modularity depends on the level of the local connectivity of communities in networks under study, while the difference between CM and ER depends on the heterogeneity in networks. To compare the behaviors of various modularity (Table 1), we conducted extensive simulations by tuning various parameters (Table 2). We use the normalized mutual information (NMI) to evaluate the performance of different modularity for detecting communities in the networks [22]. NMI can reflect the similarity between two community divisions, revealing the amount of extracted community information in a network with known community structures. NMI=1 if two community divisions are matched perfectly, and NMI<1 otherwise.

**Table 2:** Networks used in the experiments. [A, B] denotes the parameter will vary from A to B. “1.5 *k*_max_” denotes that *c*_max_ changes with *k*_max_ in the given proportion, while “0.015 *N*” denotes that the parameter varies with *N* in the given proportion. t_1_=2, t_2_=2, μ=0.2.

*Networks*		*N*	*k*_*m*_	*k*_*max*_	*c*_*min*_	*c*_*max*_
**NET1**	(a)	1000	10	10	10	[10, 100]
	(b)	1000	10	30	10	[30, 100]
	(c)	5000	10	10	10	[10, 100]
	(d)	5000	10	30	10	[30, 100]
**NET2**	(a)	1000	10	[10, 100]	10	150
	(b)	5000	10	[10, 100]	10	150
	(c)	1000	10	[10, 100]	10	1.5 *k*_max_
	(d)	5000	10	[10, 100]	10	1.5 *k*_max_
**NET3**	(a)	[1000, 10000]	10	30	10	50
	(b)	[1000, 10000]	10	30	10	150
	(c)	[1000, 10000]	10	100	10	150
	(d)	[1000, 10000]	10	0.015 *N*	10	0.020 *N*
**NET4**	(a)	5000	10	30	10	150
	(b)	5000	10	100	10	150
	(c)	5000	10	30	10	600
	(d)	5000	10	100	10	600

### Effect of community-size difference

**Fig 1:**
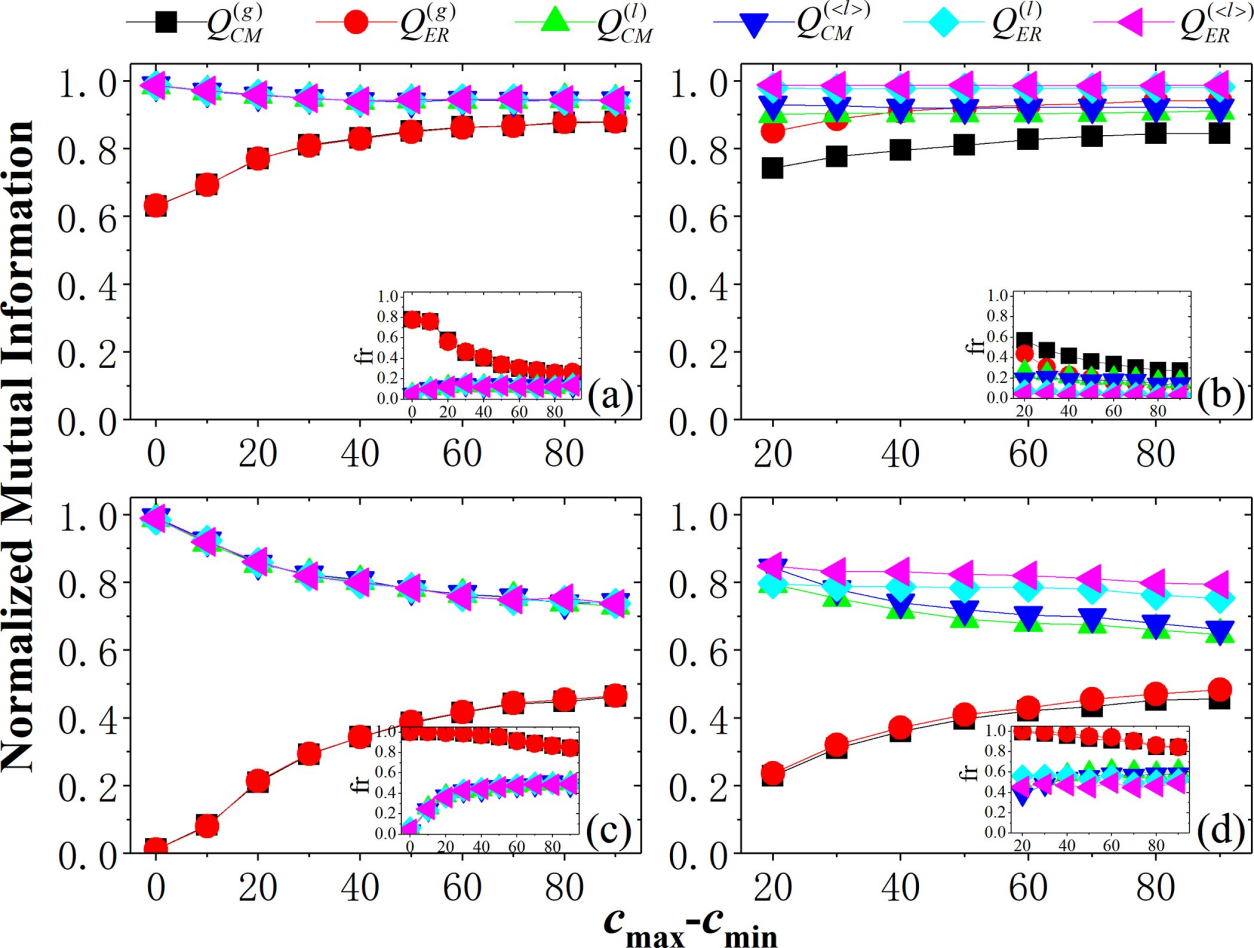
Normalized mutual information (NMI) obtained by different modularity as a function of community-size difference (*c*_min_-*c*_max_) in the NET1 networks. Parameters of networks: (a) N=1000, k_max_=10; (b) N=1000, k_max_=30; (c) N=5000, k_max_=10; (d) N=5000, k_max_=30 (see Table **1** for details of network parameters). Inset graphs show the fraction (fr) of affected nodes due to the merging of communities (i.e., the first-type resolution limit) by different methods as a function of community-size difference in the networks.

Firstly, we show the effect of community-size difference (c_min_-c_max_) on the methods (see Fig 1). In the networks, the values of NMI for most methods are less than 1. This is because some communities merge due to the first-type resolution limit of modularity with fixed resolution parameter. The inset graphs showed the fraction of nodes affected by the merging of communities, and the larger the fraction of affected nodes, the less the values of NMI. This confirmed that the methods indeed encountered the resolution limit. As we see, NMI of local modularity is larger than that of global modularity in the networks, at least for the corresponding CM or ER models. This means that the local modularity can mitigate the effect of the resolution limit and thus outperform the corresponding global modularity, because it makes use of the local connectivity of (small-size) communities. This confirmed the advantage of the local modularity with this local information.

For global modularity, NMI increases with the increase of community-size difference. This is because the appearance of large-size communities results in the decrease of the number of small-size communities, leading to the decrease of the fraction of the merged (small-size) communities by the global modularity (see inset graphs in Fig 1). For local modularity, NMI decreases with the increase of community-size difference, because the number of (small-size) communities with local connectivity decreases. Moreover, when *k*_m_=*k*_max_ (see Fig 1(A) and 1(C)), these networks are homogeneous in vertex degree, and thus the CM and ER-based null models will be equivalent. As expected, they generate similar results respectively for global or local modularity, while the heterogeneity of vertex degree will increase the difference of them.

### Effect of vertex-degree difference

**Fig 2:**
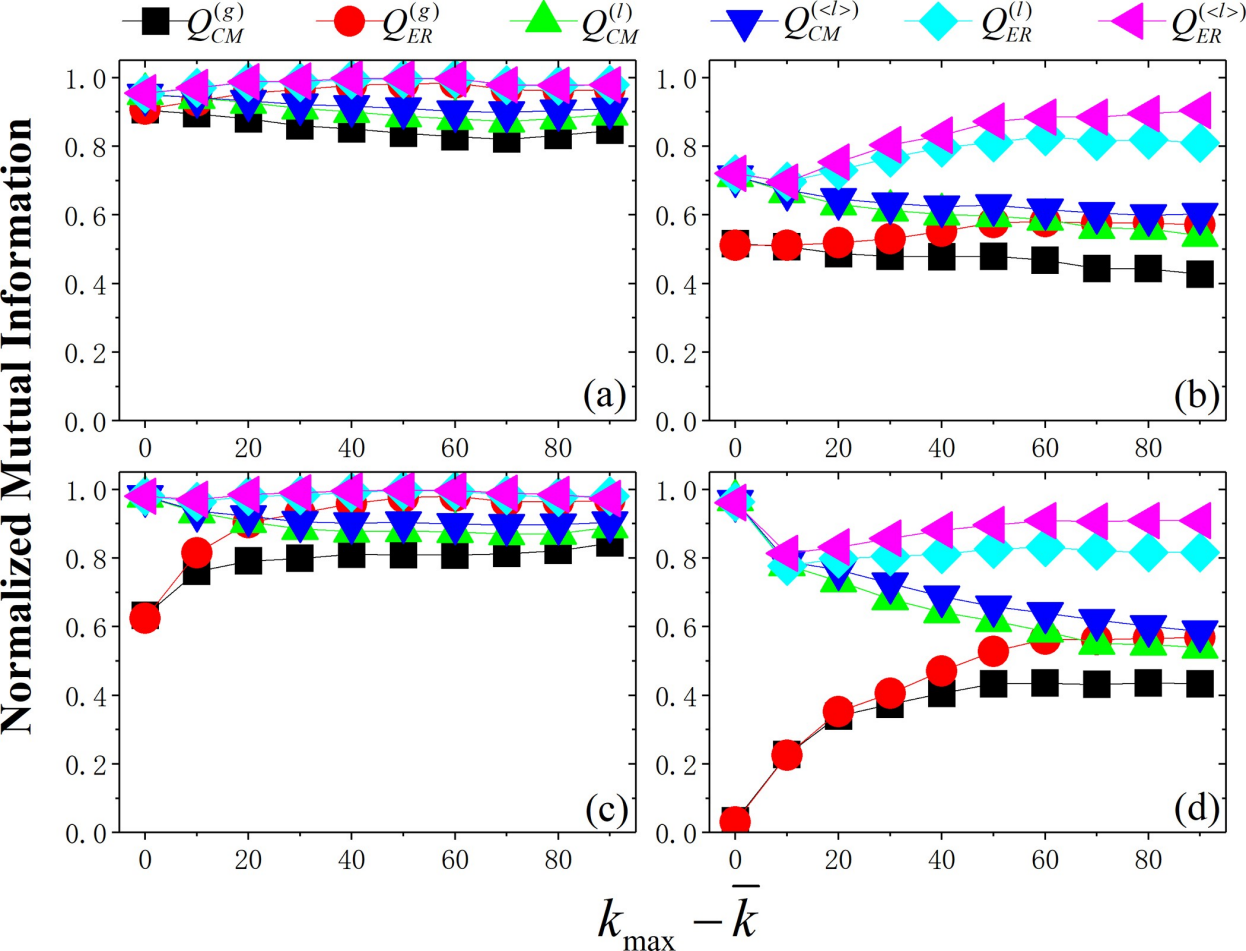
NMI obtained by different modularity as a function of vertex-degree difference (*k*_max_-*k*_m_) in the NET2 networks. Parameters of networks: (a) N=1000, c_max_=150; (b) N=5000, c_max_=150; (c) N=1000, c_max_=1.5 *k*_max_; (d) N=5000, c_max_=1.5 *k*_max_ (see Table 1 for details of network parameters).

Fig 1(B) and 1(D) show the existence of the difference of the CM and ER-based methods in the networks due to the vertex-degree difference. Further, Fig 2 shows how the vertex-degree heterogeneity affects the methods, and enlarges the difference of the methods. In small-size networks (e.g., N=1000), the curves of the methods have differences, but have also overlapping. In larger-size networks (e.g., N=5000), the phenomena is more obvious. Firstly, by fixing the heterogeneity in community size (see Fig 2(A) and 2(B)), for the CM-based methods ($Q_{CM}^{\left(g\right)}$, $Q_{CM}^{\left(l\right)}$ and $Q_{CM}^{\left(<l>\right)}$), regardless of global or local ones, NMI decreases with the vertex-degree heterogeneity, especially in large-size networks (Fig 2(B)). This may be because the vertex-degree heterogeneity disturbs them, and especially makes the number of small-size communities (note that “small size” denotes small total degree) increases, which worsens the first-type resolution limit of CM-based modularity. However, for the ER-based modularity, it is not the case. Because they make use of the mean community degree ${\bar{k}}_{s}$, instead of the community degree *k*_*s*_. And the stronger the vertex-degree heterogeneity is, the more obvious the difference between ${\bar{k}}_{s}$ and *k*_*s*_ is, and the less the links of the small-size communities to others is. This makes the ER-based modularity more quickly to disconnect small-size communities than the CM-based ones, so with the degree heterogeneity, NMI for ER increases on the whole and the ER-based methods are getting more and more different from the CM-based ones.

Then, we let the heterogeneity of vertex degree and community size vary simultaneously (see Fig 2(C) and 2(D)). In this case, the *first-type* resolution limit of all modularity is to be mitigated, because of the increase of the number of large-size communities. For the global methods ($Q_{CM}^{\left(g\right)}$ and $Q_{ER}^{\left(g\right)}$), this is the main reason that leads to the clear increase of NMI, and exceeds clearly other interference factors. However, for $Q_{CM}^{\left(l\right)}$ and $Q_{CM}^{\left(<l>\right)}$, the increase of large-size communities also weakens the local connectivity of communities, and this exceeds the other factors for them, leading to the decrease of NMI. Moreover, because of the increase of the heterogeneity of vertex degree, the difference between CM and ER becomes larger and larger, especially for global modularity.

### Effect of network size

**Fig 3:**
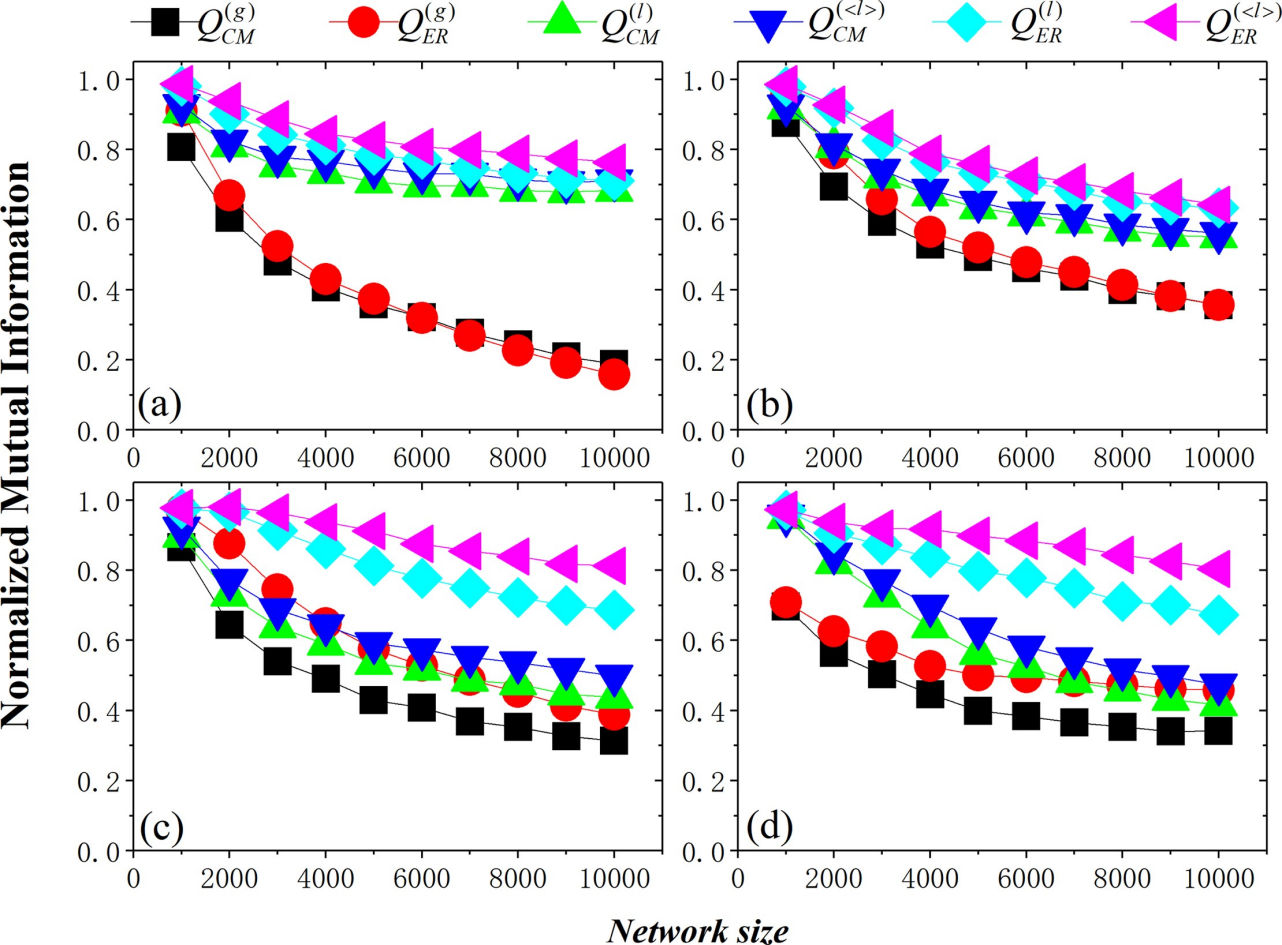
NMI obtained by different modularity as network size in the NET3 networks. Parameters of networks: (a) k_max_=30, c_max_=50; (b) k_max_=30, c_max_=150; (c) k_max_=100, c_max_=150; (d) k_max_=0.015 *N*, c_max_=0.020 *N*. (see Table 1 for details of network parameters).

Fig 3 compares the behaviors of various methods by varying the network size. For all methods, NMI decreases with the increase of network size, mainly due to the resolution limit of modularity. Increasing the vertex-degree heterogeneity will obviously increase the difference of the methods. On the whole, local modularity with fixing resolution parameter exceeds global ones, due to the use of local information. Further, by tuning the resolution parameter, the methods can better identify the underlying community structures in networks.

### Varying resolution parameter

**Fig 4:**
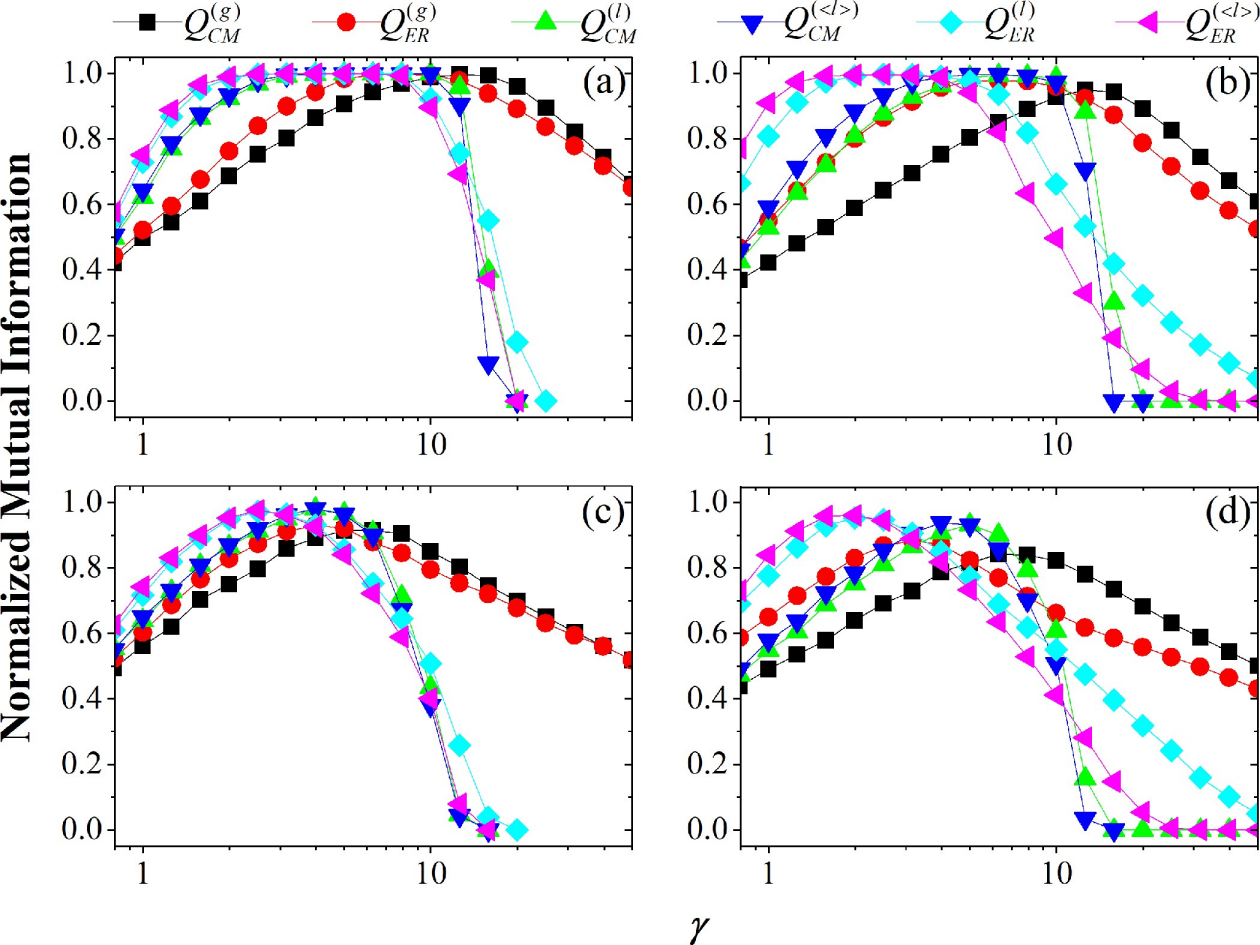
NMI of different methods as a function of *γ* in the NET4 networks with different heterogeneity of degree and community size (i.e., different values of *k*_max_ and *c*_max_). Parameters of networks: (a) k_max_=30, c_max_=150; (b) k_max_=100, c_max_=150; (c) k_max_=30, c_max_=600; (d) k_max_=100, c_max_=600. (see Table 1 for details of network parameters).

Firstly, in the networks with weak heterogeneity of community size (see Fig 4(A) and 4(B)), NMI=1 for suitable γ-values, meaning that the embedded community structures are revealed. That is to say, the first-type resolution limit of modularity has been resolved.

Secondly, the comparison between local and global modularity shows that the local modularity can reach the point of NMI=1 or the top of the curves of NMI more quickly than global ones, meaning that the local modularity can reveal the community structures more quickly, because they can earlier disconnect the small-size communities. However, the local modularity will also make the breakup of (large-size) communities more early, leading to the quicker decline of NMI.

Thirdly, in the networks with stronger heterogeneity of community sizes (see Fig 4(C) and 4(D)), because of the *second-type* resolution limit of modularity [37, 38, 44], for some methods (especially the global methods), the community structures cannot be revealed only by tuning the resolution parameter, because (large-size) communities have broken before (small-size) communities disconnect. In the cases, the local methods still have better results than global ones.

Fourthly, in the networks with larger vertex-degree heterogeneity (see Fig 4(B) and 4(D)), the difference between various methods is exhibited more clearly. For example, the ER-based methods (e.g. $Q_{ER}^{\left(<l>\right)}$ or $Q_{ER}^{\left(l\right)}$) can quicken the disconnecting of (small-size) communities, but also leads to the breakup of communities more early. By comparison, the CM-based methods (e.g. $Q_{CM}^{\left(<l>\right)}$ or $Q_{CM}^{\left(l\right)}$) delay the disconnecting of (small-size) communities, but also delay the breakup of communities. Whether there exists a kind of method by which the disconnecting of (small-size) communities can be quickened while the breakup of communities can be delayed? It is an interesting topic.

### Composite comparison in the LFR networks

**Fig 5:**
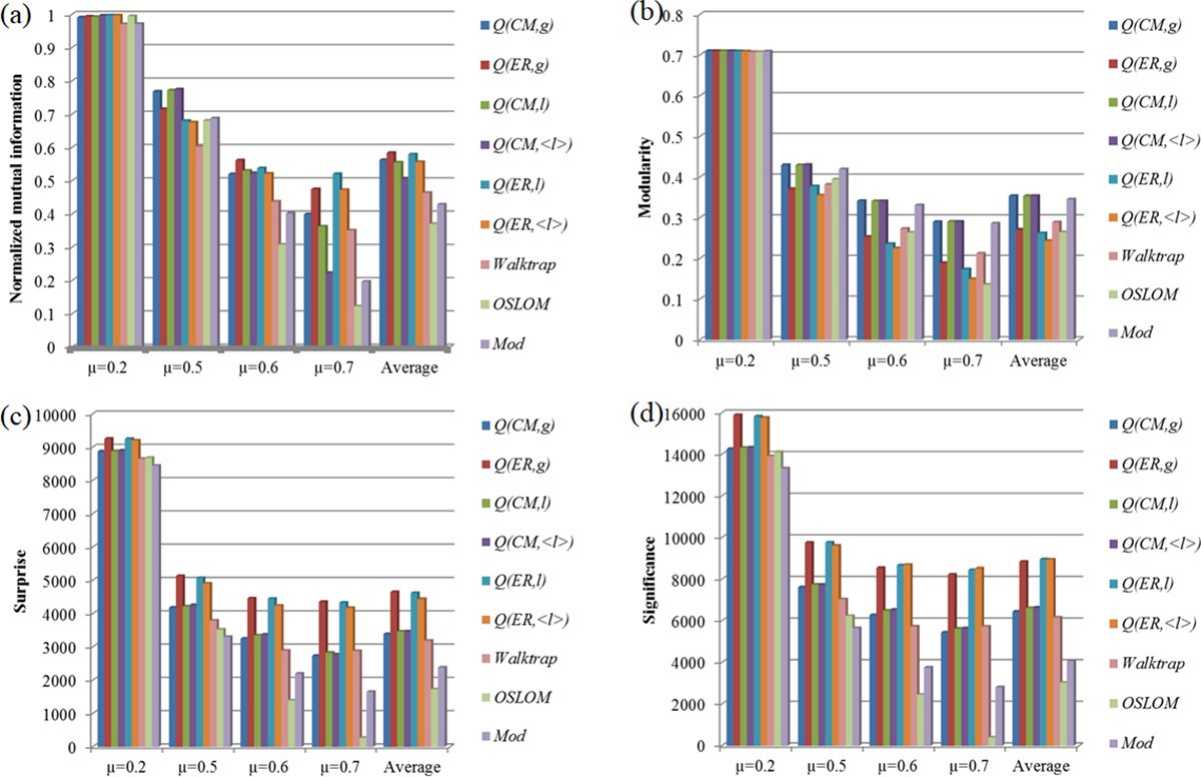
Composite comparison of different methods in the LFR networks with different μ-values. Parameters of networks: N=5000, k_m_=10, k_max_=100, ***c***_***min***_=10, and c_max_=150 (see Table 1 for details of network parameters). “Mod” denotes the original Modularity. The optimal results is given for the modularity.

Fig 5 shows the composite comparison of various methods for various μ-values in the LFR networks. By increasing value of μ, community structures will be more and more fuzzy. As a result, the NMI of the methods decreases with the increase of μ. The tunable resolution of modularity can help find the community partitions better than other methods. For example, it seems that $Q_{ER}^{\left(g\right)}$ and $Q_{ER}^{\left(l\right)}$ can generate the higher NMI than others on average.

Moreover, some statistical measures for community structures, such as Modularity [20], Surprise[19, 56–58] and Significance [59], are used to evaluate the quality of community structures especially when the real community partitions are unknown. Here, we also display the optimal values of the statistical measures for evaluating community structures by the methods, though the predefined community partitions are known in the networks (see Fig 5(B)–5(D)). For different evaluation indexes and different networks, the best results are obtained by different methods. While, according to the statistical measures, the multi-resolution modularity methods can have the ability to find the better results. For example, on average, $Q_{CM}^{\left(g\right)}$, $Q_{CM}^{\left(l\right)}$ and $Q_{CM}^{\left(<l>\right)}$ can generate the higher values of Modularity; $Q_{ER}^{\left(g\right)}$, $Q_{ER}^{\left(l\right)}$ and $Q_{ER}^{\left(<l>\right)}$ can generate the higher values of Surprise; $Q_{ER}^{\left(g\right)}$, $Q_{ER}^{\left(l\right)}$ and $Q_{ER}^{\left(<l>\right)}$ can generate the higher values of Significance.

### Real-world networks

**Fig 6:**
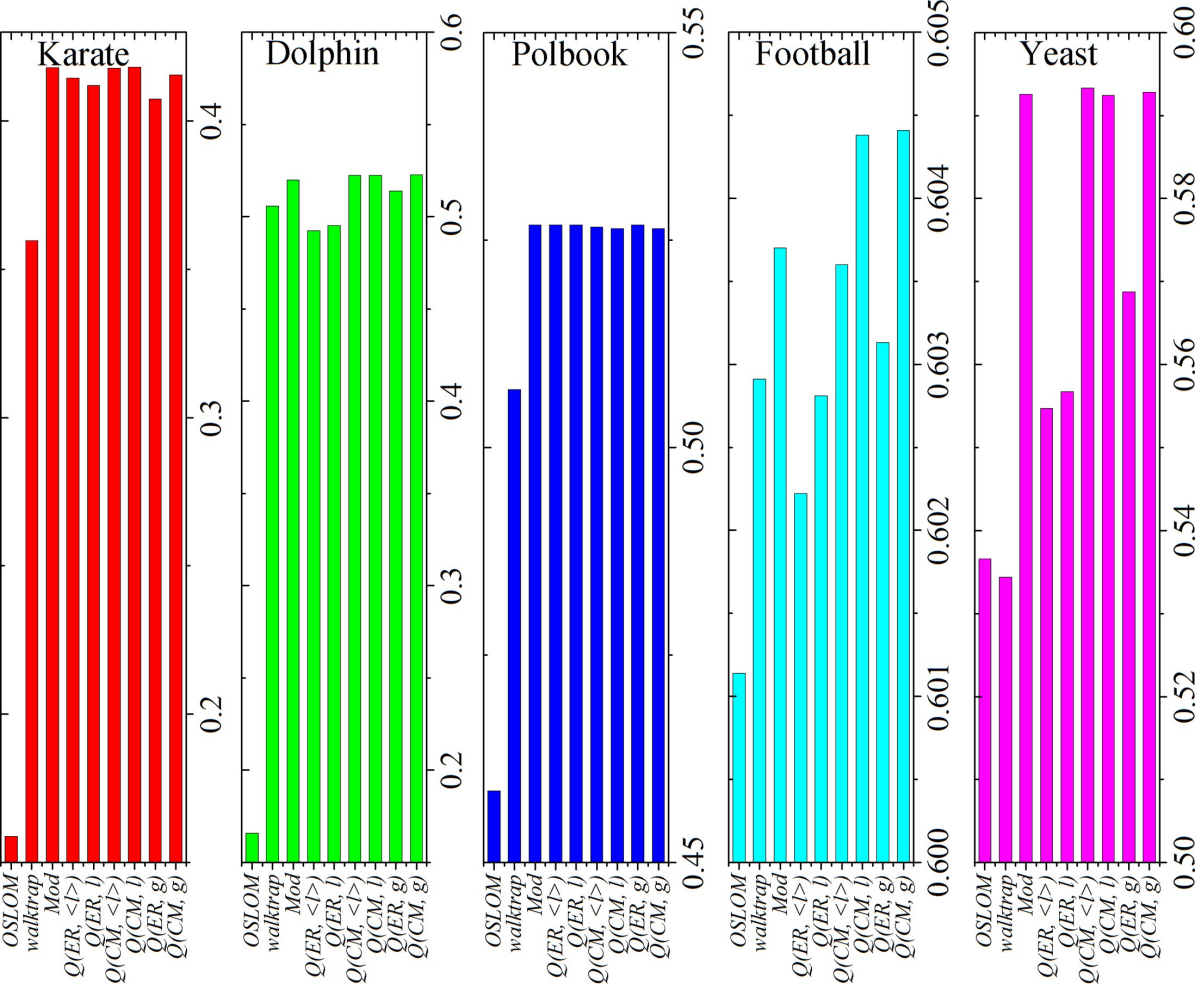
Modularity obtained by different methods in real-world networks.

Finally, the methods are applied to real-world networks. For convenience of quantitative comparison, we assess the quality of community partitions in the network by statistical approaches, Modularity, Surprise [19, 56–58] and Significance [59]. Fig 6 shows the composite results in the real-world networks.

For Modularity (see Fig 6), the (global and local) modularity-based methods can obtain high values of modularity. Especially, the four CM-based methods (*Q(CM*, *g)*, *Q(CM*, *l)*, *Q(CM*, *<l>) and* Mod) can obtain similar and relatively higher values of modularity than others in the karate, dolphin and yeast networks. In the polbook network, all the modularity-based methods can obtain similar and high values of modularity. In football network, *Q(CM*, *g)* and *Q(CM*, *l)* can obtain higher values of modularity than others.

**Fig 7:**
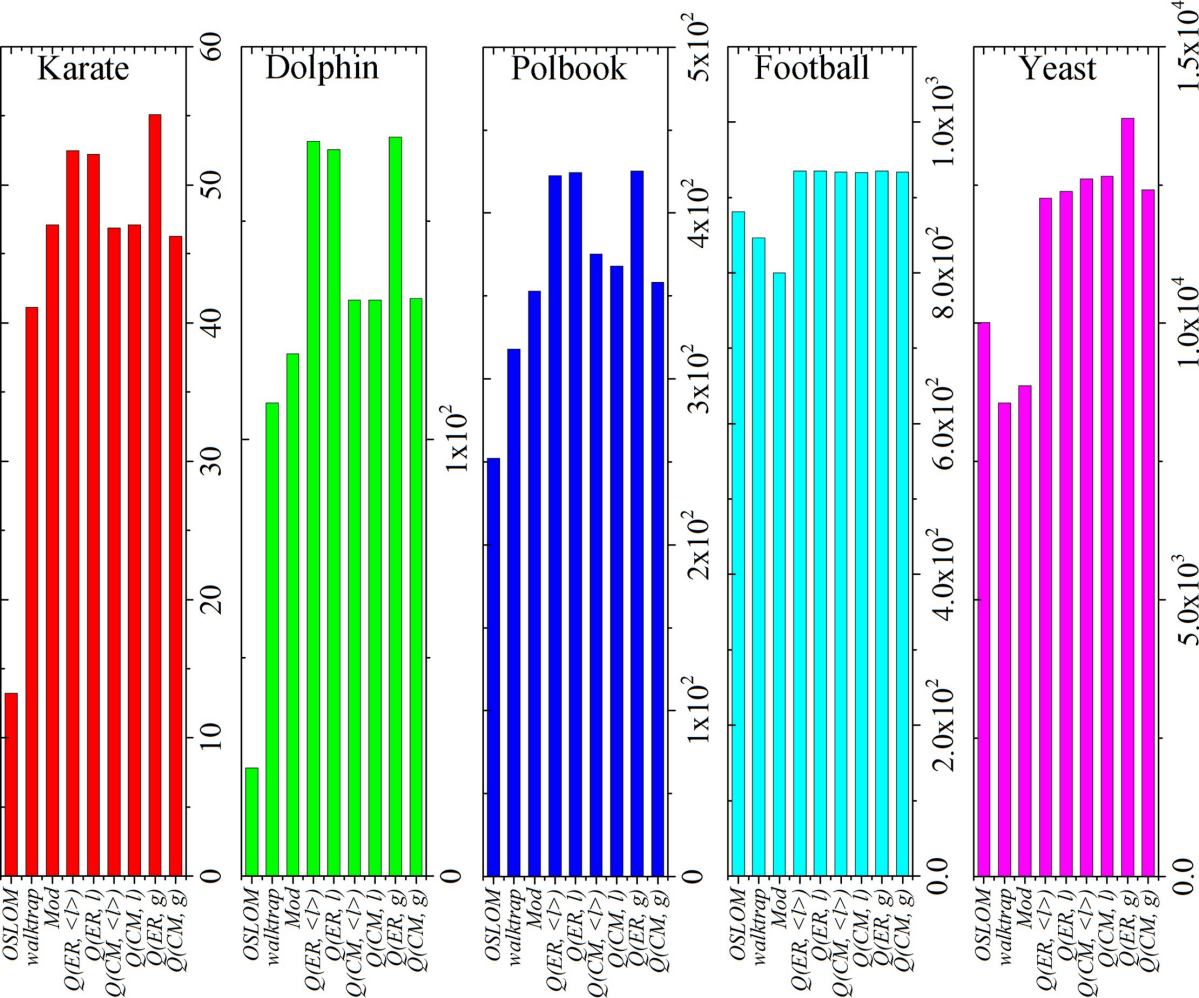
Surprise obtained by different methods in real-world networks.

For Surprise (see Fig 7), these ER-based methods (*Q(ER*, *g)*, *Q(ER*, *l)* and *Q(ER*, *<l>)*) have significantly higher values of Surprise in the karate, dolphin and polbook networks; the modularity-based methods (except original Mod) have similar results in the football network; *Q(ER*, *g)* has the best result in the yeast network.

**Fig 8:**
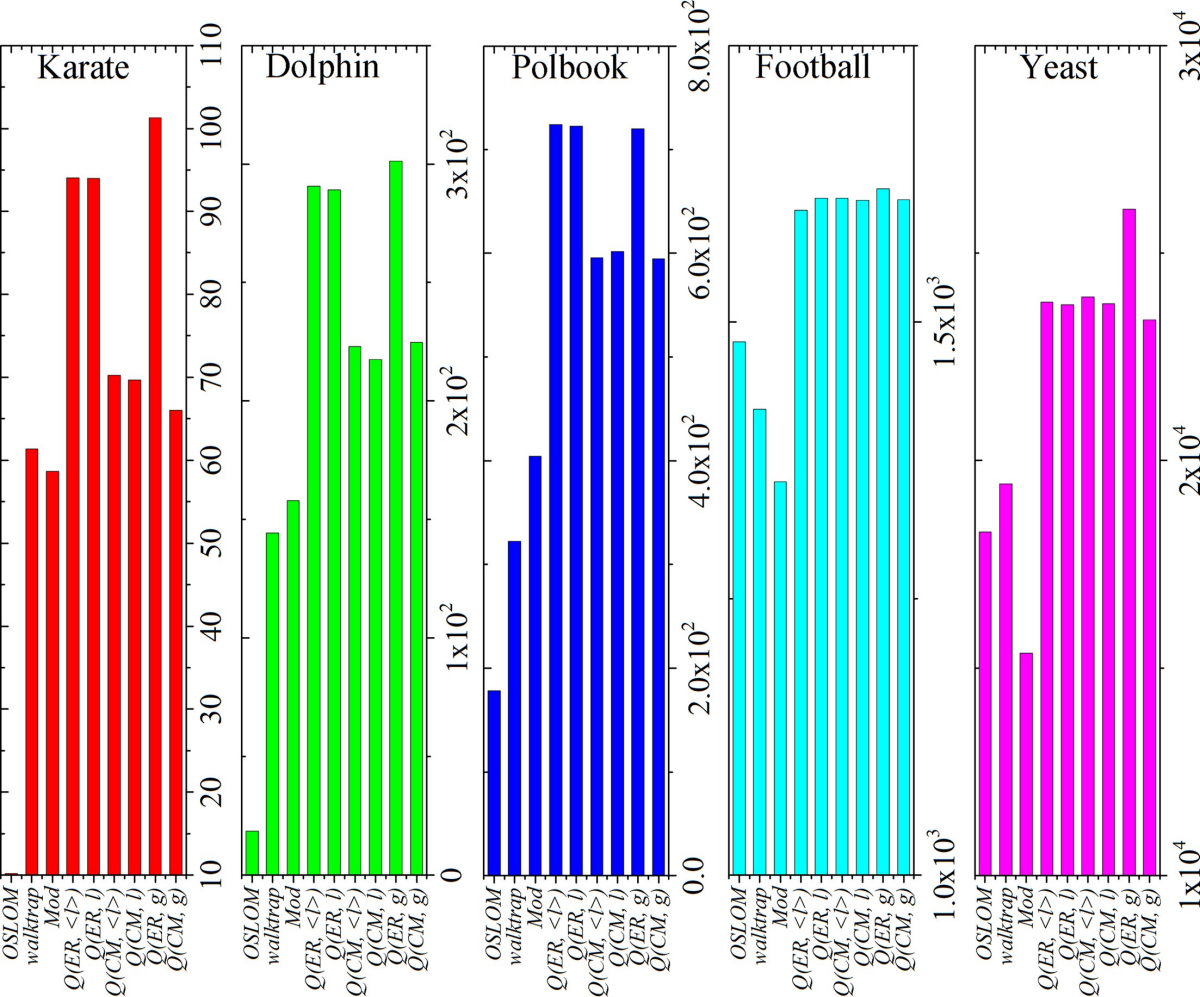
Significance obtained by different methods in real-world networks.

For Significance (see Fig 8), these ER-based methods (*Q(ER*, *g)*, *Q(ER*, *l)* and *Q(ER*, *<l>)*) can generate relatively higher values of significance in the karate, dolphin and polbook networks, while *Q(ER*, *g)* obtains the best results in the karate and dolphin networks. All the modularity-based methods (except original Mod) have similar and higher values of significance in the football and yeast networks, and especially *Q(ER*, *g)* can generate the best results in yeast network.

## Conclusion and discussion

Optimizing statistical measures for community structures is one of most important strategies for community detection in complex networks. In the paper, by using a type of *self-loop* rescaling strategy, we introduced a set of global modularity functions and a set of local modularity functions for community detection in networks. By a kind of the self-consistent method, the modularity functions are optimized for community detection.

We compared and analyzed the behaviors of the modularity-based methods in community detection. On the one hand, compared with the global modularity, the local modularity has the notable advantage, which closely depends on the *local connectivity* of communities that universally exists in the large-size networks. Particularly, the local modularity can eliminate the *first-type* limit of modularity more quickly, and can eliminate or alleviate the *second-type* limit of modularity in networks, because of the use of the *local* information in networks.

On the other hand, for the *second-type* limit of modularity, the ideal methods should be able to quickly disconnect (small-size) communities while delay the breakup of (large-size) communities. By comparing the CM and ER-based modularity, each of them exhibits one of the above properties respectively—the ER-based modularity can quicken the disconnecting of (small-size) communities, while the CM-based modularity delays the breakup of communities. This may provide a useful insight for community detection: combining various methods may be able to generate interesting results.

Systematical comparisons and analysis of community detection methods are of help for the understanding of the existing methods’ behaviors, the improvement of the methods, as well as the proposal of new methods. We give an attempt for this purpose. With regard to the modularity in the paper, the local modularity only takes into account the directly connected communities. Maybe, other factors, e.g. the connection strength between communities, can provide more useful information for community detection. Similarly, many other methods may also benefit from the use of more useful topological information. Moreover, the local modularity has advantages in general networks, but there still exist rooms for improvement. For example, if networks without the local connectivity of (small-size) communities are given, the local modularity’s advantages will disappear. In this case, the localization of communities should deserve in-depth studies further. Finally, we expect the research can enrich the knowledge for modularity optimization methods in community detection and provide useful insight into the problem of community detection in complex networks.

## Appendix A

### Self-loop rescaling strategy

The *self-loop* rescaling strategy is to rescale the network topology structure by assigning a suitable *self-loop* to each node, which can affect the null model of modularity and its weight [34]. As we know, the modularity is affected by the topology structure, the community division and the null model. Therefore, various modularity can be derived based on the Newman-Girvan modularity by *self-loop* rescaling strategy. The derived modularity can be maximized by the existing modularity optimization algorithms, which extend the application of the algorithms.

#### General modularity based on self-loop rescaling

The self-loop rescaling strategy is to assign each vertex a self-loop $\gamma_{s}\cdot k_{i}^{eff}-k_{i}$, where *γ*_*s*_, a factor of tuning the self-loop, depends on community *s* that vertex *i* belongs to, *k*_*i*_ is the degree of node *i* in the original network, $k_{i}^{eff}$ is the *effective* degree of node *i* in null model. Then, the original modularity of Newman and Girvan can be re-written as,

$$\begin{array}{l} Q(\gamma )=\frac{1}{\sum_{s}\gamma_{s}k_{s}^{eff}}\sum_{i,j}\left(A_{ij}+(\gamma_{s}k_{i}^{eff}-k_{i})I_{ij}-\frac{\gamma_{s}k_{i}^{eff}\cdot \gamma_{s}k_{j}^{eff}}{\sum_{s}\gamma_{s}k_{s}^{eff}}\right)\delta (C_{i},C_{j})\\ \;=\frac{1}{\sum_{s}\gamma_{s}k_{s}^{eff}}\sum_{s}\left(k_{s}^{in}+\gamma_{s}\cdot k_{s}^{eff}-k_{s}-\gamma_{s}^{2}\frac{{\left(k_{s}^{eff}\right)}^{2}}{\sum_{s}\gamma_{s}k_{s}^{eff}}\right)\\ \;=\frac{\beta -1}{\beta }+\frac{1}{\beta }\cdot \frac{1}{2M}\sum_{s}\left(k_{s}^{in}-\gamma \frac{{\left(k_{s}^{eff}\right)}^{2}}{2M_{s}^{eff}}\right) \end{array},$$ (2)

where *M* is the total number of edges in the network, $k_{s}^{in}$ is the inner degree of community *s*, *k*_*s*_ is the total degree of community *s*, *γ* is a tunable resolution parameter; *I*_*ij*_ is the identity matrix; $\sum_{}k_{i}^{eff}\cdot \delta (C_{i},s)=k_{s}^{eff}$ is the total *effective* degree of group *s*, and $\sum k_{s}^{eff}=2M$. *γ*_*s*_ is discussed below. Please see Table 1 for details of $k_{i}^{eff}$ and $M_{s}^{eff}$.

For global modularity, the parameters of the self-loop rescaling are very simple: $M_{s}^{eff}=M$, *γ*_*s*_ = *γ* and $k_{i}^{eff}=k_{i}^{}$ for CM-based modularity ($k_{i}^{eff}=\bar{k}$ for ER-based modularity). For local modularity, the rescaling scheme is a little complicate, because we need special treatment to constrain $M_{s}^{eff}$ to expected forms. In order to consider the local connectivity of communities, let,

$$\gamma_{s}^{2}\frac{{\left(k_{s}^{eff}\right)}^{2}}{\sum_{s}\gamma_{s}k_{s}^{eff}}=\gamma \frac{{\left(k_{s}^{eff}\right)}^{2}}{2M_{s}^{eff}}.$$ (3)

The right-hand side in the equation is the expected form in null model, while the left-hand side is the original expresion from the self-loop rescaling. Here, we need to find suitable *γ*_*s*_ so that the two sides of the equation are equal. To get the value of *γ*_*s*_, some tricks are used. By Eq (3),

$$\gamma_{s}^{}k_{s}^{eff}=\sqrt{\gamma \sum_{s}\gamma_{s}k_{s}^{eff}}\frac{k_{s}^{eff}}{\sqrt{2M_{s}^{eff}}}.$$ (4)

By summing Eq (4) over all communities and suitable transformation, we obtain,

$$\sum_{s}\gamma_{s}k_{s}^{eff}=\gamma {\left(\sum_{s}\frac{k_{s}^{eff}}{\sqrt{2M_{s}^{eff}}}\right)}^{2}=2M\beta ,$$ (5)

where $\beta =\frac{\gamma }{2M}{\left(\sum_{s}\left(\frac{k_{s}^{eff}}{\sqrt{2M_{s}^{eff}}}\right)\right)}^{2}$ (for simplification). By substituting Eq (5) into Eqs (3) or (4), we obtain the final expression,

$$\gamma_{s}^{}=\gamma \frac{1}{\sqrt{2M_{s}^{eff}}}\sum_{s}\frac{k_{s}^{eff}}{\sqrt{2M_{s}^{eff}}}.$$ (6)

Here $k_{s}^{eff}$ is determined by the form of $k_{i}^{eff}$ in the self-loop rescaling, and $M_{s}^{eff}$ is determined by the local connectivity of community and expected null model (see **Table 1** for details). For example, in $Q_{CM}^{\left(l\right)}$, $M_{s}^{eff}$ is the total number of links in community s and the neighborhood of it, while $k_{i}^{eff}=k_{i}^{}$. By Eq (6), one can specify the self-loop to get expected modularity. Moreover, the self-loop rescaling for global modularity can be regarded as a special case of the above scheme. For global modularity, $M_{s}^{eff}=M$.

By combining the self-consistent optimization for the local modularity, the factors before the summation in *Q*(*γ*) is independent of the optimization procedure for given *γ*-value and community partition. So the (multi-resolution) *modularity* based on the self-loop rescaling is equivalent to the modularity in text.

#### Analysis of resolution of modularity based on self-loop rescaling

As discussed previously, the modularity has resolution limit, and the resolution inequality of the Newman-Girvan modularity can be denoted as *k*_*s*_*k*_*t*_<2*M*⋅*e*_*st*_, where *k*_*s*_ is the total degree of community, *e*_*st*_ is the number of links between communities, and *M* is the total number of links in the network [26]. If the inequality is satisfied, the communities cannot be identified by the modularity. The self-loop rescaling can change the relative size of communities, so as to change the resolution of modularity. For example, by assigning each vertex *i* a self-loop *α*⋅*k*_*i*_ (where *α* is a parameter and *k*_*i*_ is the original degree of vertex), the degree of community changes to be (1+*α*)*k*_*s*_, and thus the above inequality changes to be *k*_*s*_*k*_*t*_<2*M*⋅*e*_*st*_/(1+*α*). Increasing *α*-value can makes the inequality more difficult to be satisfied. So more (small-size) communities can be revealed by increasing the *α*-value. By adjusting the *α*-value, one may discover communities at different levels.

### Self-consistent optimization for local modularity

Similarly to the global modularity, communities in networks can be revealed by optimizing the above local modularity. Here, because of the self-containing property of local modularity based on the self-loop strategy, we have proposed a self-consistent optimization for the local modularity, which is inspired by the self-consistent field theory in physics.

The self-consistent method is the basic iterative method for solving the complicate equations in quantum mechanics. The basic idea is to first give an estimate of the solution according to a certain method, and then use this estimate to calculate the related parameters to get an improved estimate. The process is repeated to improve the estimate until it becomes stable. We use similar strategy to optimize the local modularity based on self-loop rescaling. We first give an initial community division, and then use it to calculate the neighborhood of communities and the related parameters of self-loop rescaling. Then we run the modularity-optimization algorithm in the rescaled network to get improved community division. Here, the Louvain algorithm is used in the rescaled network, which is a kind of fast and efficient algorithm for modularity optimization [17], though any effective algorithms for maximizing modularity can be used in principle.

The optimization procedure for local modularity needs an initial community assignment. There are two simple choices: all vertices are assigned into one group and each vertex is given an independent community label. In detail, the general optimization procedure for the local modularity is as following.

- Give the value of *γ* and initial community assignment of nodes in the network under study.
- Calculate the *γ*_*s*_-values by the community division and assign the new self-loop to each vertex.
- The network with the new self-loop is re-divided into communities by the optimization algorithm.
- Repeat from step (b) until community partition is unchanged or the iteration time is larger than a maximal iteration time.

There exist sub-optimal community partitions in many networks [24]. Scanning the search space to find the optimum of modularity is NP-hard in a large network. As a result, it is difficult to find an exact and consistent partition for each *γ-*value. Thus, a maximal iteration time (*Tm*) is set for the self-consistent optimization. If the value of modularity is unchanged, the self-consistent algorithm is terminated. If the iteration time *T* > *Tm* but the value of modularity is still unstable, the algorithm is also terminated and the partition of the *T*^*th*^ iteration is outputted as the final partition.

For the local modularity, when the resolution parameter varies, the scale of the community structure will change accordingly. With the increase of *γ*, they can detect the community structures from macro- to micro-scales. When *γ*→0, they will assign all nodes into a single and large community. The network will split into a set of single-node communities, each of which only contains one node, if *γ* is very large.

### Assessment standards

For networks with known community structures, Normalized mutual information (NMI) [22] is used to evaluate the performance of methods; for networks with unknown community structures, Surprise [19, 56–58] and Significance to select a suitable resolution parameter and evaluate the quality of found partitions.

#### Normalized mutual information

Normalized mutual information (NMI) evaluates the similarity between two community divisions[22]. NMI can reflect the amount of extracted community information correctly by different methods in networks with known community structures. NMI=1 if two partitions are matched perfectly, and zero otherwise. The value of NMI will decrease with the decrease of the matching. So NMI can evaluate the performance of methods in community detection.

#### Surprise

*Surprise* is a statistical approach to assess the quality of a community partition in network, with higher values corresponding to better partitions[19, 56–58]. It was shown that *Surprise* can give better characterization for community structures in networks than modularity in several complex benchmarks. Given a community partition in a network, based on cumulative hyper-geometric distribution, *Surprise* is defined as the minus logarithm of the probability of observing the number of intra-community links or more in Erdös-Rényi graphs,

$$Surprise=-\log\sum_{j=m_{\mathrm{int}}}^{\min(m,M_{\mathrm{int}})}\frac{\left(\begin{array}{c} M_{\mathrm{int}}\\ j \end{array}\right)\left(\begin{array}{c} M-M_{\mathrm{int}}\\ m-j \end{array}\right)}{\left(\begin{array}{c} M\\ m \end{array}\right)},$$ (7)

where *M* denotes the maximal number of all possible links in a network; *M*_int_ denotes the maximal number of possible intra-community links of the given partition; *m* denotes the number of existing links in the network; while *m*_int_ denotes the number of existing intra-community links in the partition.

### Significance

Similar to Surprise, significance is a recently proposed measure for estimating the quality of community partitions[59], which evaluates the possibility that dense communities occur in random graphs. The definition of it is,

$$Significance=\sum_{s}\left(\begin{array}{c} n_{s}\\ 2 \end{array}\right)D(p_{s}|p),$$ (8)

Here the sum runs over all communities; *p*_*s*_ is the density of links within the community; *p* is the density of links in the network; Kullback-Leibler divergence is *D*(*p*_*s*_|*p*) = *p*_*s*_log(*p*_*s*_/*p*)+(1−*p*_*s*_)log(1−*p*_*s*_)/(1−*p*)). Significance could be used to choose resolution parameters so as to determine suitable community partitions, and cloud also be directly optimized as objective function to find the optimal community partitions.

## Supporting information

S1 File The real-world networks used in the work. (ZIP)
